# A Disintegrin and Metalloproteinase 10 (ADAM10) Is Essential for Oligodendrocyte Precursor Development and Myelination in the Mouse Brain

**DOI:** 10.1007/s12035-022-03163-0

**Published:** 2022-12-23

**Authors:** Dazhi Guo, Fei Huang, Ruijun Xue, Yuehong Ma, Lin Xiao, Huifang Lou, Shuyi Pan

**Affiliations:** 1 Department of Hyperbaric Oxygen, 6Th Medical Center of PLA General Hospital, Beijing, 100048 China; 2 Department of Stomatology, 6Th Medical Center of PLA General Hospital, No. 6, Fucheng Rd, Haidian District, Beijing, 100048 China; 3 Department of Hyperbaric Oxygen, School of Medicine, South China University of Technology, Guangzhou, 510006 China; 4 Institute for Brain Research and Rehabilitation, South China Normal University, Guangzhou, 510631 China; 5 Institute of Neuroscience, University of Zhejiang, Hangzhou, 310058 China

**Keywords:** A disintegrin and metalloproteinase 10 (ADAM10), Myelination, Oligodendrocyte precursors (OPCs), Central nervous system (CNS), Notch-1

## Abstract

A disintegrin and metalloproteinase 10 (ADAM10) plays an essential role in the regulation of survival, proliferation, migration, and differentiation of various neural cells. Nevertheless, the role of ADAM10 in oligodendrocyte precursors (OPCs) and myelination in the central nervous system (CNS) of developing and adult mouse brains is still unknown. We generated ADAM10 conditional knockout (ADAM10 cKO) mice lacking the ADAM10 gene primarily in OPCs by crossing NG2-Cre mice with ADAM10 ^loxp/loxp^ mice. We found that OPCs expressed ADAM10 in the mouse corpus callosum and the hippocampus. ADAM10 cKO mice showed significant loss of back hair and reduction in weight and length on postnatal (30 ± 2.1) day, died at (65 ± 5) days after birth, and exhibited the “anxiety and depression-like” performances. Conditional knockout of ADAM10 in OPCs resulted in a prominent increase in myelination and a decrease in the number of OPCs in the corpus callosum at P30 owing to premyelination and lack of proliferation of OPCs. Moreover, the number of proliferating OPCs and mature oligodendrocytes (OLs) also decreased with age in the corpus callosum of ADAM10 cKO mice from P30 to P60. Western blot and RT-PCR results showed that the activation of Notch-1 and its four target genes, Hes1, Hes5, Hey1, and Hey2, was inhibited in the corpus callosum tissue of ADAM10 knockout mice. In our study, we provided experimental evidence to demonstrate that ADAM10 is essential for modulating CNS myelination and OPC development by activating Notch-1 signaling in the developing and adult mouse brain.

**Supplementary Information:**

The online version contains supplementary material available at 10.1007/s12035-022-03163-0.

## Introduction

Myelination is essential for ensuring fast and efficient propagation of action potentials along axons and providing metabolic trophic support for axons [1]. In the central nervous system (CNS), the development of the myelin sheath requires a fun-tuning of processes, including differentiation of oligodendrocyte precursors (OPCs) to oligodendrocytes (OLs), OL selection of target axons, maturation of OLs, and finally synthesis and assembly of myelin proteins [2, 3]. OPCs, also known as NG2 cells, are CNS-resident stem cells that are generated in restricted areas such as the subventricular zone (SVZ), and subsequently proliferate and migrate throughout the CNS during embryonic development [4]. Some OPCs differentiate into OLs, while the others remain in a slowly proliferative or quiescent state, which accounts for 5–10% of nerve cells in the adult CNS. Upon opportune physiological or pathologicalstimuli, this part of OPCs can respond to increased proliferation and subsequent differentiation into myelinating OLs, which are the primary cell source of remyelination in the CNS [5]. Broad mechanisms regulate OPCs development and myelination, including transcription factor, chromatin modifications, and external signals including thyroid hormone, neurotrophic factors, and oxygen saturation and mechanotransduction [6, 7].

ADAM10 is a member of the disintegrins and metalloproteinases (ADAMs) family, comprising disintegrin, metalloprotease, cysteine-rich, and epidermal growth factor (EGF)–like domains [8]. ADAM10 knockout mice are only 2/3 of the size of normal mice, and usually die around 9.5 days of embryonic period (E9.5) due to obvious developmental defects in the cardiovascular system and CNS [9]. In CNS, ADAM10 is predominantly expressed in neurons in areas such as the cerebral cortex, hippocampus, thalamus, and cerebellar granular cells [10]. It was also observed that ADAM10 mRNA was sparingly expressed in OLs in numerous fiber tracts at later embryonic stages [11]. Growing evidence suggests that ADAM10 regulates the survival, proliferation, migration, and differentiation of various neural cells, including axonal outgrowth and myelination by Schwann cells in PNS [10, 12]. In a cuprizone-induced demyelination model, overexpression of ADAM10 can suppress demyelination and reduces seizure susceptibility by generating a soluble amyloid precursor protein fragment (sAPPα) to ease neuroinflammation and oxidative stress [13]. However, the physiological role of ADAM10 in myelination and OPC development in the developing and adult mice brain remains unclear.

In this study, we first detected the expression pattern of ADAM10 in the white matter of the mouse brain using in situ hybridization (ISH) and immunohistochemical staining. Next, we crossed NG2-Cre mice (Cre is specifically expressed in OPCs) with ADAM10 ^loxp/loxp^ mice with conditional knockout of ADAM10 in OPCs and investigated the role and mechanism of ADAM10 on OPCs development and myelination in the CNS. Our results showed that ADAM10 was expressed in OPCs in the mouse corpus callosum and hippocampus. ADAM10 cKO mice showed significant loss of back hair and reduction in weight and length on postnatal (30 ± 2.1) day, died at (65 ± 5) days after birth, and exhibited the “anxiety and depression-like” performances. Conditional deletion of ADAM10 resulted in premature myelination and failure of OPCs to proliferate, ultimately leading to demyelination. We also found that the activation of Notch-1 and its four target genes, Hes1, Hes5, Hey1, and Hey2, was inhibited in the corpus callosum tissue of ADAM10 knockout mice, suggesting that ADAM10 is essential for modulating CNS myelination and OPC development by activating the Notch-1 signaling pathway in the developing and adult mouse brain.

## Materials and Methods

### Generation of ADAM10 Conditional Knockout Mice and Determination of the Genotype

All animal experimental protocols were approved by the Animal Ethics Committee of the Sixth Medical Center of PLA General Hospital. ADAM10 ^loxp/loxp^ mice (from Prof. Duan) were crossed with heterozygous NG2-Cre mice (Jackson Lab) to generate ADAM10 ^loxp/−^and NG2-Cre^+/−^ mice were bred with ADAM10 ^loxp/loxp^ mice to produce ADAM10 cKO (homozygous ADAM10 ^loxp/loxp^; NG2-Cre^+/−^) offspring. Genotypes were determined by PCR on genomic DNA extracted from mouse tails, as previously described. PCR primers used were as follows: ADAM10 (5′-ACC TCT TAG CGA TAC CAC AAG CC-3′ and 5′-CCA TGG AAG TGT CCC TCT TCA TTC GTA GG-3′) and actin (5′-GAG CAC CCT GTG CTG CTC ACC GAG G-3′ and 5′-GTG GTG GTG AAG CTG TAG CCA CGC T-3).

### In Situ Hybridization and Immunohistochemistry

The method for generation of the ADAM10 cRNA probe and ISH was described previously [10]. The ADAM10 cRNA probe is labeled with digoxigenin and the primary antibody is alkaline phosphatase conjugated with anti-digoxigenin antibodies. Alkaline phosphatase developed with NBT/BCIP. The positive color was purple-blue. Following ISH, the sections were blocked with 10% bovine serum albumin (Beyotime Institute of Biotechnology, Haimen, China) for 1 h at room temperature, and post-hybridized slides were incubated with anti-RNA binding protein, NG2 antibody (1:200; Chemicon, Rolling Meadows, IL, USA) overnight at 4 °C. Three washes for 10 min with 0.01 M phosphate-buffered saline (PBS), after incubating with horseradish peroxidase (HRP)–conjugated rabbit anti-mouse (1:1,000; Gene Tech, Shanghai, China; cat. no. GP016129) secondary antibodies for 1 h at room temperature and then washed twice for 10 min in 0.01 M PBS. Color development was performed using a DAB kit (OriGene Technologies, Inc., Beijing, China), according to the manufacturer’s protocol.

### Behavioral Test

**Self-grooming:**
  The mouse was placed into clear observation cylinders before manual recording of self-grooming for 4 h by two highly trained observers using the grooming analysis algorithm. A grooming “bout” was characterized as self-grooming without interruption (defined as a full stop in grooming for more than 3 s).
**Open field test (OFT):**
  The mouse was placed in the center of a 44 × 44 × 44-cm opaque plexiglass arena and recorded for 10 min using an overhead HDMI camera with Open Broadcast Software (OBS). Mouse tracking was automated using ANY MAZE software (Stoelting). The average speed, distance traveled, and time immobile graphs were generated using SigmaPlot (SysStat Software Inc.).
**The elevated plus maze (EPM):**
  The mouse was placed in the center of the maze, which consisted of two open arms (6 cm × 32 cm) and two closed arms (6 cm × 32 cm with 19 cm tall opaque walls) with a center area of 6 cm × 6 cm and 54 cm above the floor. The surrounding room was dark, and the maze was lit with overhead lights. The mouse faced an open arm and was allowed to explore for 5 min, and the movement and time spent in the closed arms were recorded automatically using the ANY MAZE animal video analysis system. The maze was cleaned with 70% ethanol before each trial.
**(4) Tail suspension test (TST):**
  The mouse was suspended by its tail more than 10 cm above the floor for 6 min. The ANY MAZE video analysis system was used to detect periods and times of complete immobility. Mice that climbed their tails or fell off the hanger were excluded from analysis.

### Electron Microscopy

The mice were anesthetized with 4% chloral hydrate, exposed the heart, cannulated the left ventricle, and cut open the right atrial appendage. First, flushed quickly with warm normal saline and then perfused the heart with a mixed fixative (1.25% glutaraldehyde and 4% paraformaldehyde in phosphate buffer). The brain was removed and post-fixed in 2% glutaraldehyde + 4% paraformaldehyde in 0.1-M cacodylate buffer (pH 7.2) at 4 °C for 24 h. After fixation, the corpus callosum was trimmed and fixed again, rinsed with 1% osmic acid, dehydrated with gradient concentration acetone, embedded in epoxy resin, and then subjected to semi-thin and ultra-thin sections, and observed by transmission electron microscopy (CM-12; FEI) coupled with a camera and its application software (Eagle 2 K × 2 K; FEI).

### FluoroMyelin Green Fluorescent Myelin Stain

Mouse brains were removed and fixed in 4% paraformaldehyde in phosphate-buffered saline (PBS) for 24 h at 4 °C before cryoprotection in 30% sucrose. Frozen 20-μm sections were rinsed with phosphate-buffered saline for at least 20 min and then incubated with FluoroMyelin Green fluorescent myelin staining (1:300; Molecular Probes, Eugene, OR, USA) for 20 min at room temperature. The sections were mounted onto slides using mounting medium (Vector Laboratories, Burlingame, CA, USA).

### Western Blot Assay

Proteins were prepared using standard protocols. For protein blotting, antibodies against ADAM10 (Cell Signaling, 14,194, 1:500), MBP (Abcam ab7349, 1:1500), proteolipid protein (PLP) (Abcam,ab105784, 1:1000), Notch1 (Invitrogen, MA1-81,888, 1:500), Notch1 intracellular domain (NICD, Cell Signaling Technology, CST4147, 1:1000), and β-actin (Proteintech 66,009–1,1:5000) were used. Antibody binding was detected using horseradish peroxidase–conjugated secondary antibodies (Abbkine), followed by an ECL kit (Zeta LIFE). The intensity was normalized to the β-actin level and expressed as a relative fold change relative to the control.

### Quantitative RT-PCR

Total RNA was extracted from the brain using TRIzol reagent, following the manufacturer’s protocol. Real-time PCR of 2-μl cDNA was used to determine the expression levels of Hes1, Hes5, Hey1, and Hey2 on an ABI 7500 Real-time PCR System with SYBR Premix Ex Taq mix (TaKaRa) in 20-μl reaction volumes. Relative levels of transcripts were calculated by normalizing to β-actin and wild-type (WT) mice (the mean expression of the WT mice was set to 100%) using the 2 − △△CT method. The primer sequences used are listed in Table 1.

**Table 1:** Primers used in real-time RT-PCR analysis

Primer	Sequence (5′–3′)	Product length
Hes1-F	TCAACACGACACCGGACAAAC	155 bp
Hes1-R	ATGCCGGGAGCTATCTTTCTT	
Hes5-F	CAGCCCGTAGAGGACTTTCTT	103 bp
Hes5-R	GCAGTTCCGCCTTCACAA	
Hey1-F	CCGACGAGACCGAATCAATAAC	125 bp
Hey1-R	TCAGGTGATCCACAGTCATCTG	
Hey2-F	AAAAGGCGTCGGGATCGAATA	177 bp
Hey2-R	AGCATGGGCATCAAAGTAGCC	
β-actin-F	TACGCCAACACAGTGCTGTCTG	200 bp
β-actin-R	CTGCTTGCTGATCCACATCTGC	

### Culture of Mouse OPCs

Primary mouse OPCs were derived and cultured using the method previously described by Guo et al. [14]. The cell suspension was plated on a poly ornithine–coated plate in an OPCs medium to achieve OPC proliferation for 1–3 days, followed by immunocytochemistry.

### Immunohistochemistry and Immunocytochemistry

Immunohistochemistry and immunocytochemistry were performed according to the routine methods. Dilutions of the primary antibodies were as follows: rat anti-MBP (1:200; SAB2108749, Sigma-Aldrich), rabbit anti-Olig2 (1:200; 387R-1, Sigma-Aldrich), rabbit anti-NG2 (1:200; SAB5700198, Sigma-Aldrich), mouse anti-NG2 (1:300, 14–6504-82, Sigma-Aldrich), monoclonal antibody CC1 (1:200, 17–0661-82, Thermofisher Scientific), rabbit anti-PDGFa (1:500, PA5-50,565, Thermofisher Scientific), mouse monoclonal anti-Ki67 antibody (1:200, MIB1, SANTA CRUZ), polyclonal anti-ADAM10 (1:100; ZRB2367; Sigma-Aldrich), anti-GFAP (1:200, MA5-12,023, Thermofisher Scientific), anti-NeuN (1:100, 66,836–1-Ig, Proteintech), and anti OX42 (1:300, ab33827, Abcam). Samples were incubated with corresponding primary antibodies overnight at 4 °C, followed by rinsing in PBS, and stained with Cy3 (1:1000; Invitrogen, Carlsbad, CA, USA) or Alexa Fluor 488-conjugated secondary antibodies (1:1000; Invitrogen) for 1 h at room.

### Fluorescence Microscopy

A confocal microscope (Fluoview 1000; Olympus; or Fluoview 500; Olympus) was used to acquire fluorescence images, which were taken with a resolution of 1024 × 1024. The gain of the photomultiplier was adjusted to maximize the signal-to-noise ratio without causing image saturation. Image processing and analysis were performed using Image-Pro Plus5.1 (Media Cybernetics, Shanghai, China).

### Statistical Analysis

Statistical analysis was performed using the GraphPad Prism 6.00 software (La Jolla, CA, USA). Data are expressed as mean ± SEM. At least three pairs of WT and ADAM10 cKO littermates were used in each experiment. All groups were compared using a two-tailed unpaired Student’s *t*-test, unless otherwise specified. A probability value of *P* < 0.05 indicated a significant difference.

## Results

### OPCs Express ADAM10 mRNA

**Fig. 1:**
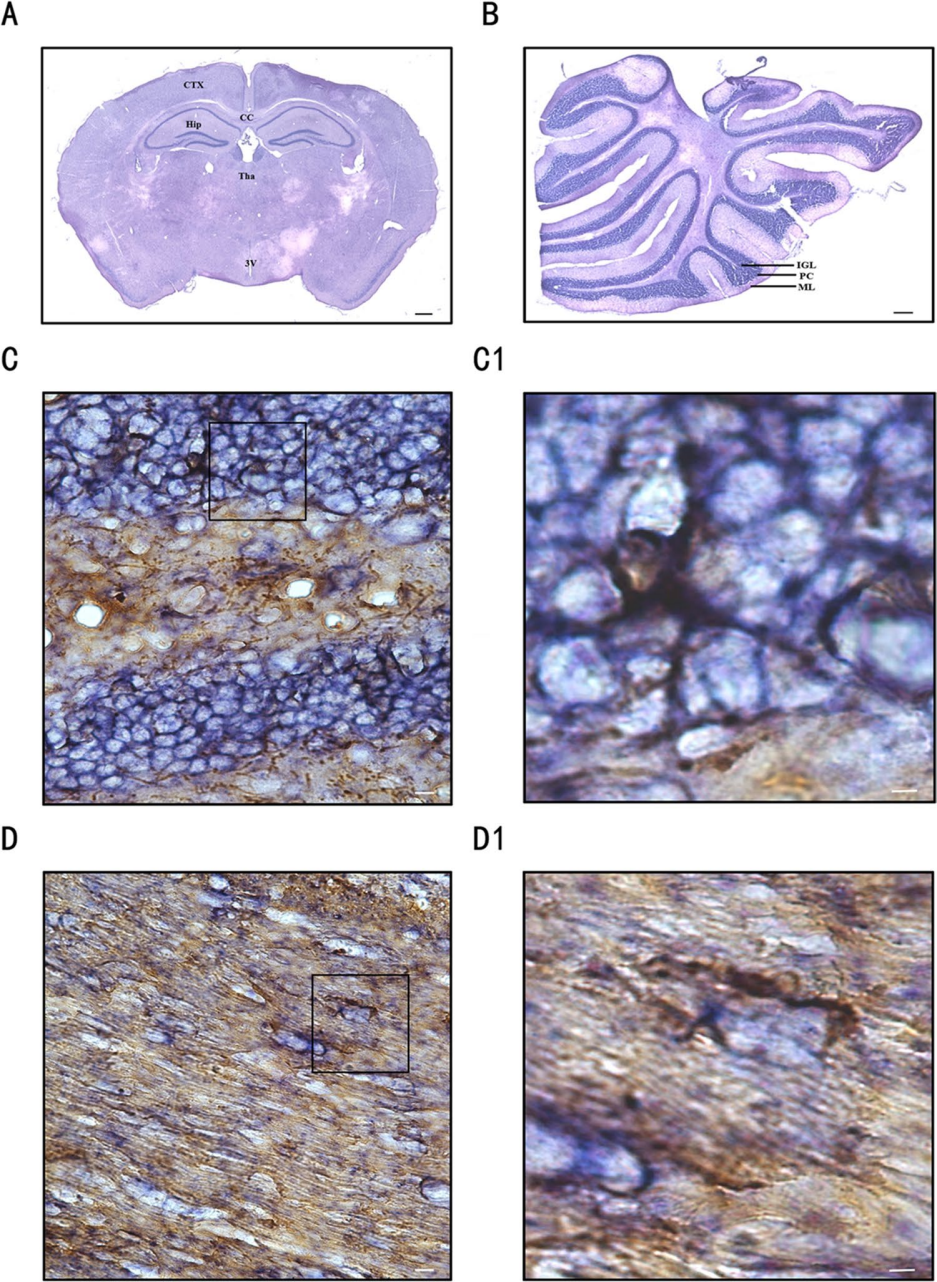
Identification of the ISH-positive OPCs in the brain of adult mice. (**A**, **B**) In situ hybridization results of ADAM10 cRNA probe in adult mouse cerebral coronal section (**A**) and cerebellum (**B**). Scale bar, 500 µm. CTX, cortex; CC, corpus callosum; Hip, hippocampus; Tha, thalamus; 3 V, the third ventricle. (C, C1, D, D1) Double staining results of ADAM10 in situ hybridization and NG2 immunostaining in the corpus callosum (C, C1) and hippocampal dentate gyrus (D, D1). Scale bar, 20 µm. (C1, D1) Zoom box of C and D. Scale bar in zoom box, 8 µm. ADAM10, a disintegrin and metallopeptidase domain 10; NG2, Neuron-glial antigen 2 or chondroitin sulfate proteoglycan 4

To determine whether ADAM10 is expressed in OPCs, combined ISH and immunohistochemical staining with an NG2 antibody (an OPC-specific marker) was used. Alkaline phosphatase and NBT/BCIP staining (blue) were used to identify ADAM10 expression, and HRP-labeled immunohistochemical staining (brown) was used to label OPCs. OPCs expressing ADAM10 were stained blue purple. Consistent with previous studies [10], ADAM10 mRNA expression was predominantly distributed in the gray matter area where neurons were concentrated, including the cerebral cortex, hippocampus, thalamus, and cerebellar granular cells in the adult mouse CNS (Fig. 1A, B). However, double staining indicated that there were several ADAM10 ISH-positive cells coexpressing NG2 in the white matter area, including the corpus callosum and dentate gyrus of the hippocampus (Fig. 1C, D).

### ADAM10 Is a Conditional Ablation in OPCs

**Fig. 2:**
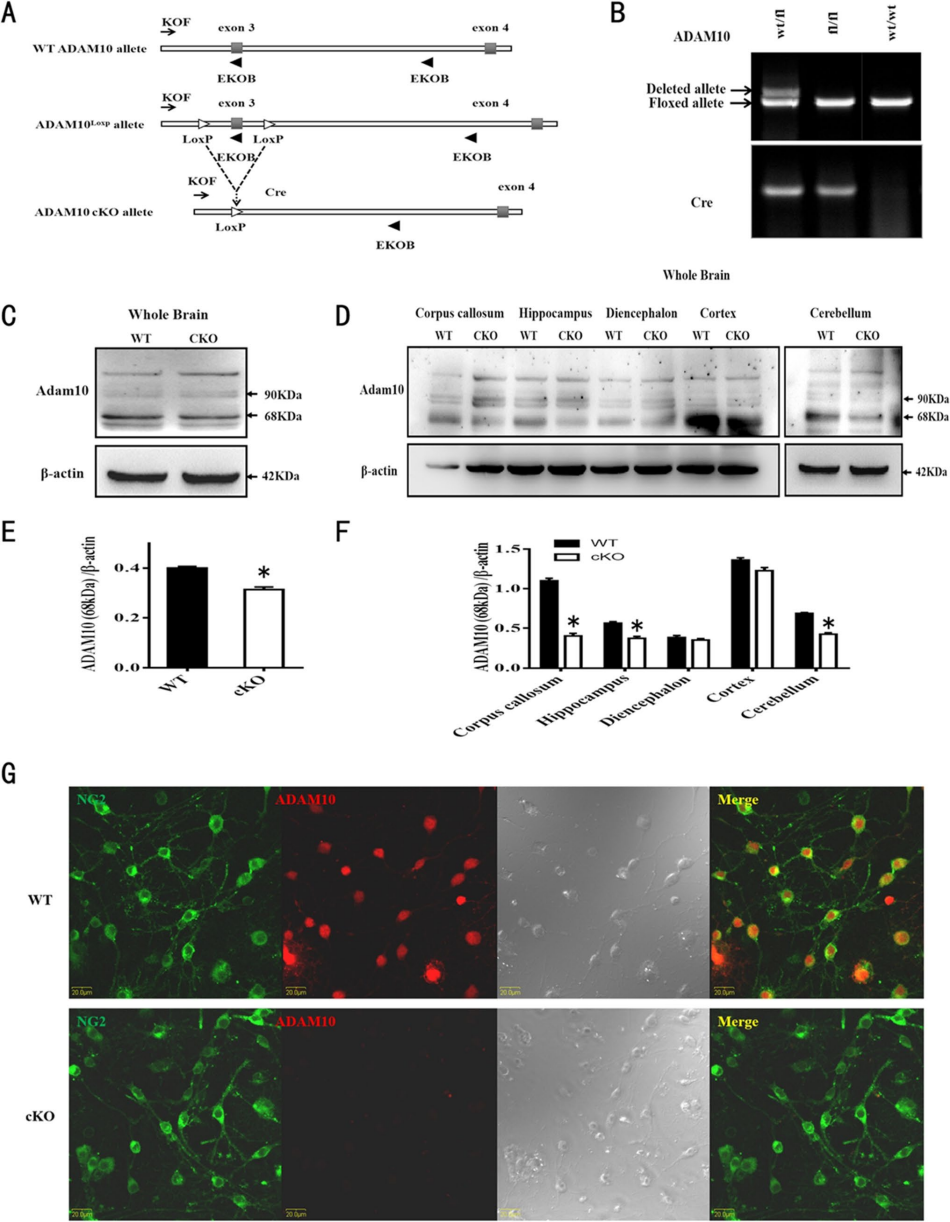
ADAM-10 is conditional ablation in OPCs. (**A**) A schematic representation of the genomic the wide-type (WT), the floxed, and the recombined ADAM10 allele (cKO allele). Gray box: exon. Open arrowheads: loxP. Solid arrowheads: primers. (**B**) PCR analysis of genomic tail DNA of wild type (wt/wt), heterozygous (wt/fl), or mutant (fl/fl) mice using primer sets that detect the wild-type and floxed ADAM10 alleles as well as the Cre gene. (**C**, **D**) Detection of ADAM10 proteins from the lysate of the whole brain, cortex, hippocampus, diencephalon, corpus collasum, and cerebelum of cKO and WT mice at P60 by Western blot. (E, F) Quantitative analysis of the active, mature 68 kDa ADAM10 expression levels. *n* = 3 mice for each group. Data represent mean ± SEM. *p < 0.05. (**G**) Labeling of OPCs from wild-type (WT) and ADAM10 cKO mice using the antibodies to ADAM10 (red) and NG2 (green). Scale bar, 20 µm

We generated OPCs conditional knockout ADAM10 mice by inserting two loxP sites in the cis orientation into the third exon of the ADAM10 gene (Fig. 2A). OPC-specific deletion of ADAM10 was obtained by crossing NG2–Cre transgenic mice with ADAM10 ^loxp/loxp^ mice [15], and PCR analysis of mouse tail DNA was used to genotype NG2-Cre/ADAM10^loxp/loxp^ (referred to ADAM10 cKO mice). PCR analysis of genomic tail DNA of wild-type (wt/wt), heterozygous (wt/fl), or ADAM10 cKO (fl/fl) mice showed that wild-type mice could detect the floxed alleles and deleted alletes (exon3 transcript) but not the Cre gene, whereas heterozygous mice could detect the Cre gene, flox, and delete alletes, whereas ADAM10 cKO mice could detect the Cre gene but not delete alletes (Fig. 2B). Western blotting showed that the levels of the active, mature 68 kDa ADAM10 protein were slightly decreased in the whole brain of ADAM10 cKO mice, but there was no significant difference from WT mice, due to the lower expression of ADAM10 in OPCs compared with other neural cells (Fig. 2C, E). When comparing the expression of ADAM10 in the cortex, hippocampus, diencephalon, corpus collasum, and cerebellum of WT and ADAM10 cKO mice, we found that there was a significant decrease in ADAM10 expression in the hippocampus, corpus collasum, and cerebellum of ADAM10 cKO mice, which are the main distribution areas of white matter (Fig. 2D, F). Moreover, co-immunostaining with anti-NG2 and anti-ADAM10 antibodies showed that the OPCs from ADAM10 cKO mice did not express ADAM10 in vitro, further suggesting that ADAM10 is conditionally ablated in OPCs (Fig. 2G).

### ADAM10 cKO Mice Exhibit the “Anxiety and Depression-Like” Performances

**Fig. 3:**
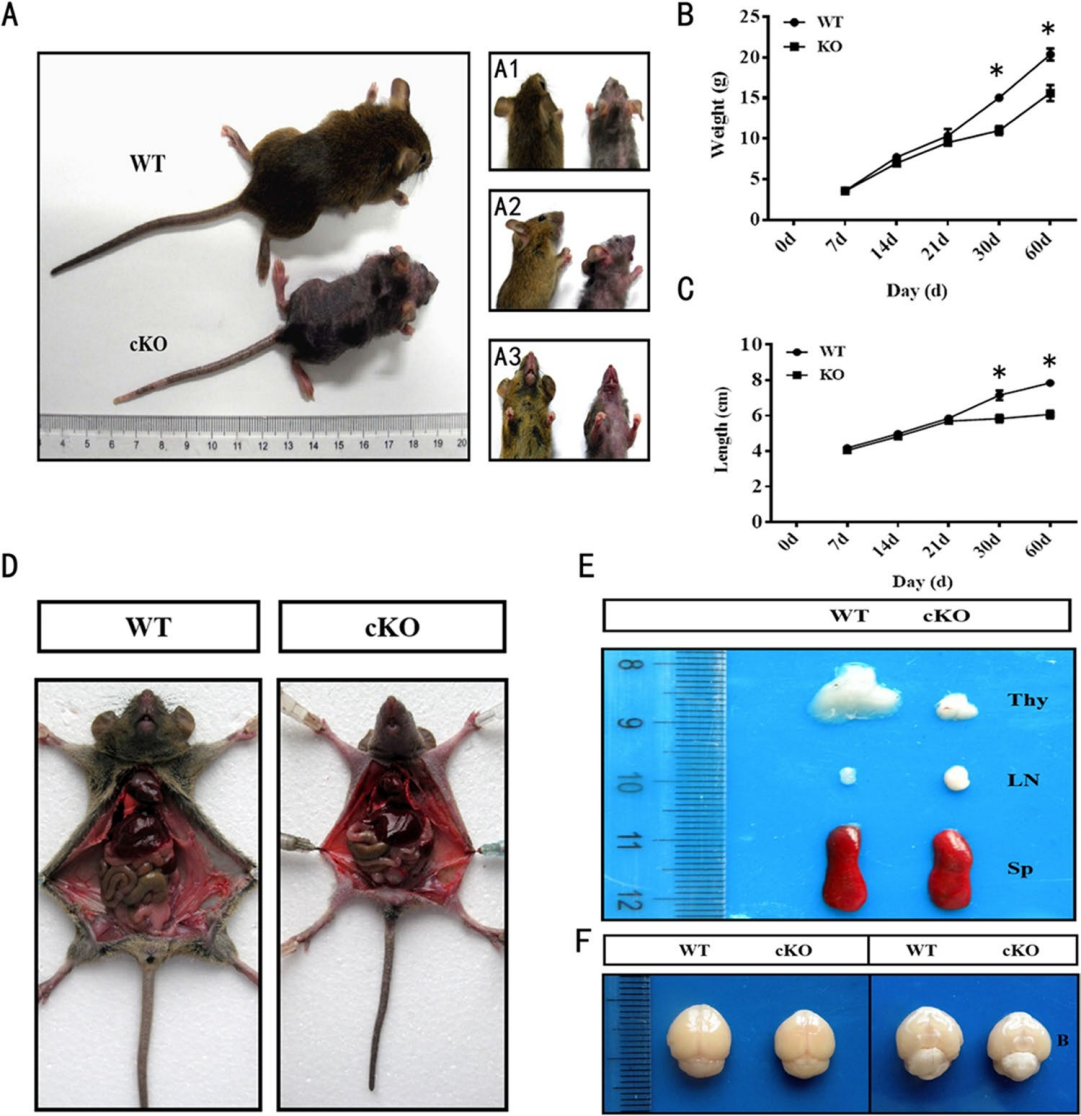
Phenotype of ADAM10 cKO mice. (**A**) Representative phenotype of ADAM10 cKO mice at P60. (A1) Top view; (A2) bottom view, and (A3) side view. ADAM10 cKO mice show significant loss of back hair and reduction in weight and length in comparison with control littermates. (**B**, **C**) Growth trends of body weight and length in ADAM10 cKO mice. ADAM10 cKO mice began to show reduction in weight and length after the postnatal 21 day, and there was significantly difference from postnatal 30 day in comparison with control littermates. (**D**, **E**, **F**) Topical anatomical comparison of organs in ADAM10 cKO mice and WT mice. Compared with wild-type mice, the brain and thymus of ADAM10 cKO mice were smaller, and the abdominal lymph nodes and spleen were larger. Thy, thymus; LN, lymph nodes; Sp, spleen; B, Brain. Data represent mean ± SEM. *p < 0.05

Compared with control littermates, ADAM10 cKO mice began showing a significant loss of back hair and reduction in weight and length on the postnatal day (30 ± 2.1), and finally died on (65 ± 5) days after birth (Fig. 3A–C). Further dissection revealed that the brain and thymus of ADAM10 cKO mice were significantly smaller, the abdominal lymph nodes and spleen were larger, and the intestinal smooth muscle was thicker (Fig. 3D–F).

**Fig. 4:**
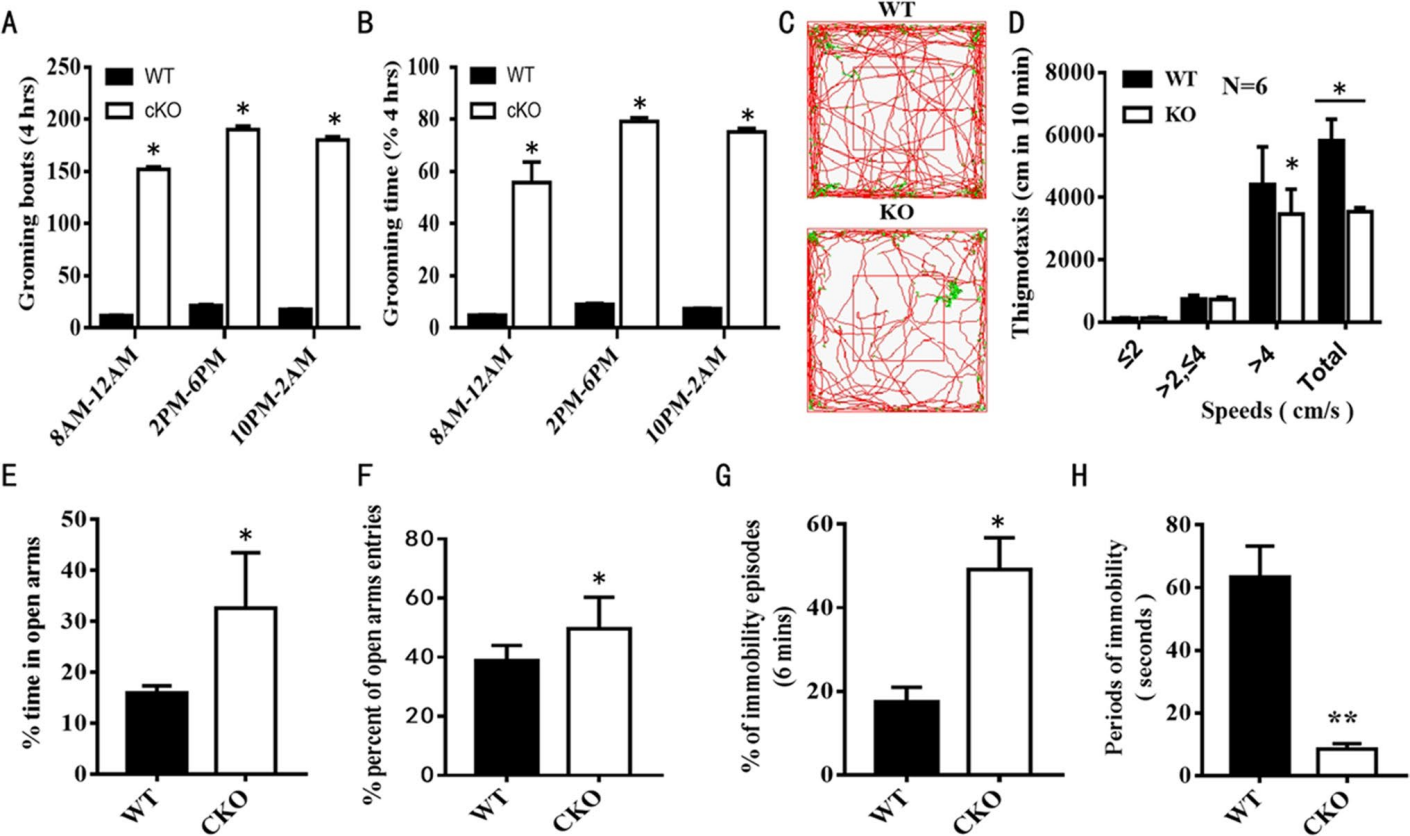
ADAM-10 cKO mice exhibit the “anxiety and depression-like” manifestations. (**A**, **B**) Mice grooming behavior analysis on P60, the total numbers of grooming bouts (**A**) and the percent of total grooming duration (s) (**B**) were measured by manual recording self-grooming of ADAM-10 cKO and WT mice for 4 h in the morning, afternoon, and evening, respectively. Regardless of the time period, ADAM10 cKO mice had more grooming bouts and higher percentages of grooming duration than WT mice. (**C**) Movements tracking of mice are recorded over 60 min in the OFT. ADAM10 cKO mice displayed a reduction in overall exploratory behavior as evidenced by the sparser pattern of exploratory paths and long duration stop in the cenral area. (**D**) Quantitative analysis of the cumulative distance traveled and the traveled distance in very low speed (≤ 2 cm/s, immobile), low speed (≤ 4 cm/s, > 2 cm/s), and high speed (> 4 cm/s). The cumulative distance and that in high speed traveled were significantly reduced in ADAM10 cKO mice. (E, F) Quantitative analysis of the percentage of time spent on the open arms (E) and open arms entries (**F**) in the EPM test. Compared with WT mice, the percent of time spent on the open arms of the maze and the percent of open arms entries were increased in ADAM10 cKO mice. (**G**, **H**) Quantitative analysis of percent of immobility episodes (**G**) and immobility periods (H) for 6 min in TST. ADAM10 cKO mice showed significantly increased percent of immobility episodes and immobility periods. *n* = 6 mice for each group, Data represent mean ± SEM. *p < 0.05

Long-term video recording revealed that ADAM10 cKO mice exhibited increased self-grooming and tremor-like behavior that might be linked to the pathogenesis of anxiety (Fig. 4A, B, Supplementary video 1, 2). Therefore, a series of behavioral tests relevant to anxiety and depression were performed on ADAM10 cKO mice. In the OFT, we observed spontaneous exploratory behavior of ADAM10 cKO and WT mice over 60 min. We found that the exploratory paths were sparser, and the stop duration was longer in ADAM10 cKO mice than in WT mice (Fig. 4C). We quantified this by analyzing the cumulative distance traveled in 10 min and found that it was significantly reduced in ADAM10 cKO mice by (33.3 ± 2.1)%. Likewise, the average speed was also reduced by (28.7 ± 1.8)%, and time immobile (speed ≤ 2 cm/s) was increased by (3.7 ± 0.5) times (Fig. 4D). In the EPM test, compared with WT mice, the percentage of time spent in the open arms of the maze and the percentage of open arm entries (% OAE) were increased in ADAM10 cKO mice, suggesting that ADAM10 cKO mice have anxiety-like properties (Fig. 4E, F). Previous results suggested that enhanced anxiety can predispose mice to depression-like behavior under some conditions [16]. We therefore performed the TST, which is frequently used in studies of depression. ADAM10 cKO mice showed a significantly increased percentage of immobility episodes (*t* =  − 4.668, df = 6, *p* = 0.023) (Fig. 4G). Moreover, the immobility periods were significantly longer in ADAM10 cKO mice (*t* =  − 14.905, df = 4, *p* = 0.031) (Fig. 4H). Taken together, these behavioral results indicate that ADAM10 cKO mice have obvious “anxiety and depression.”

### Conditional Deletion of ADAM10 in OPC Results in Premature Myelination and Failure of Myelin Maintenance

**Fig. 5:**
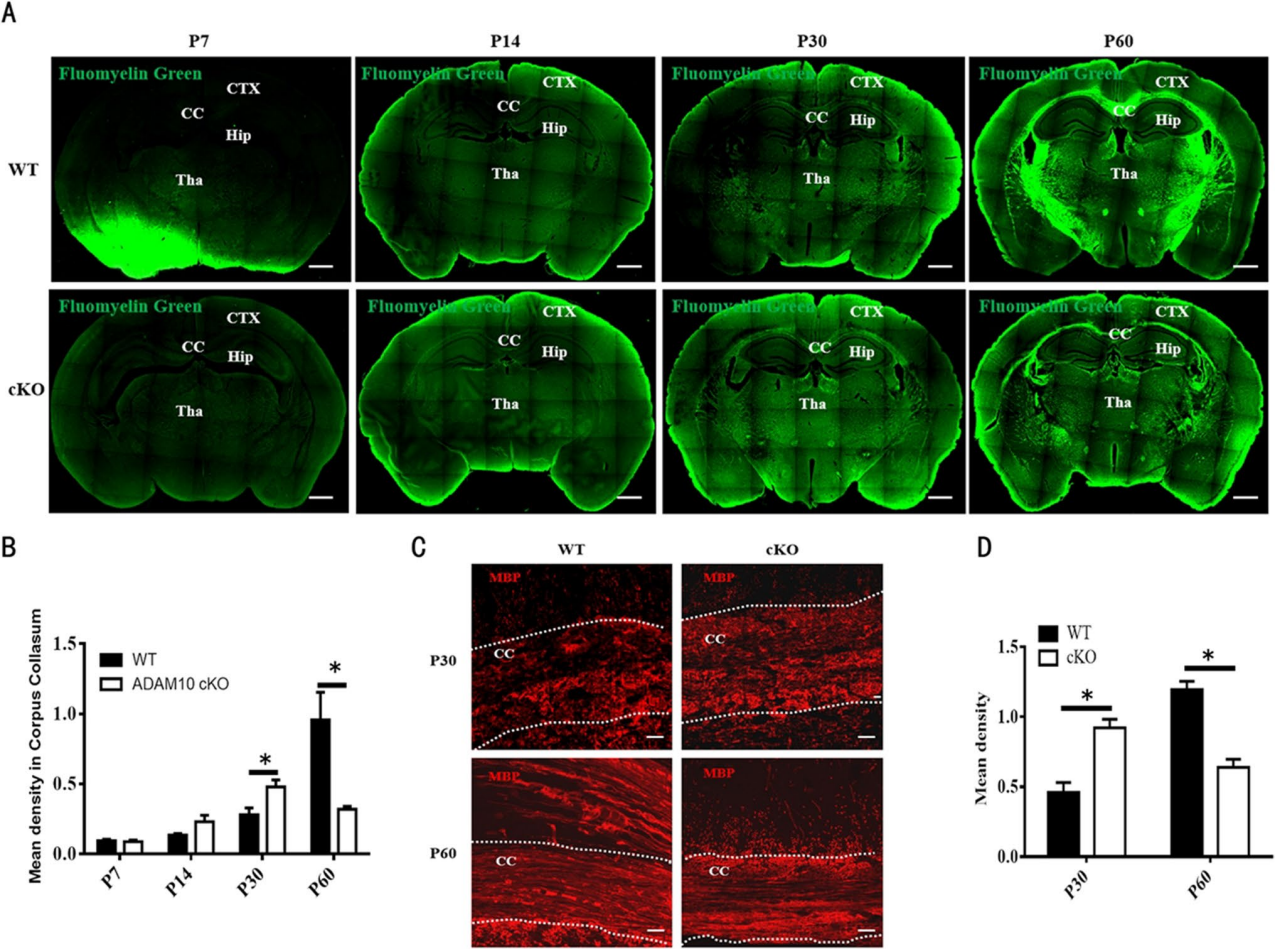
Deletion of ADAM10 in OPCs causes myelination deficits. (**A**) FluoroMyelin Green fluorescent myelin staining for myelin injury (green) at P7, P14, P30, and P60. Bar: 200 μm. (**B**) Quantitative analysis of mean density for FluoroMyelin Green fluorescent myelin staining in corpus collasum at P7, P14, P30, and P60. *n* = 3 slides from three animals per group, Data represent mean ± SEM, *p < 0.05. (**C**) Immunostaining of MBP in corpus callosum at P30 and P60. CC, corpus callosum. Scale bar: 200 μm. (**D**) Quantitative analysis of mean density for MBP staining in CC at P30 and P60. *n* = 3 slides from three animals per group, Data represent mean ± SEM, *p < 0.05

Mood disorders such as anxiety and depression are common features of demyelinating diseases [17, 18]. Previous results suggest that myelination of axon tracts within the CNS occurs throughout the first 30–40 days after birth in rodent animals [2, 19]. We assessed myelin formation in the brains of WT and ADAM10 cKO mice on P7, P14, P30, and P60 using FluoroMyelin Green fluorescent myelin staining. In WT mice, FluoroMyelin Green positive signals (green) began to appear on P30 in the corpus callosum, thalamus, and other white matter areas, and there was a gradual increase in the density of FluoroMyelin Green staining from P30 to P60. However, in ADAM10 cKO mice, positive signals began to appear on P14 in the corpus callosum, thalamus, and other white matter areas, and the density of FluoroMyelin Green (green) significantly increased at P30 and subsequently decreased at P60. Compared with WT mice, the density of FluoroMyelin Green (green) was higher on P30, but weaker on P60 in ADAM10 cKO mice, suggesting that conditional deletion of ADAM10 in OPC results in premature, but maintenance failure of CNS myelination (Fig. 5A, B). To further investigate the influence of ADAM10 on myelination in the brain, we examined the expression of the mature OL marker MBP by immunohistochemistry and western blotting. Compared with WT mice, the immunofluorescence intensity of MBP was stronger in the corpus callosum of ADAM10 cKO mice at P30, while it was weaker in the corpus callosum at P60 (Fig. 5C, D).

**Fig. 6:**
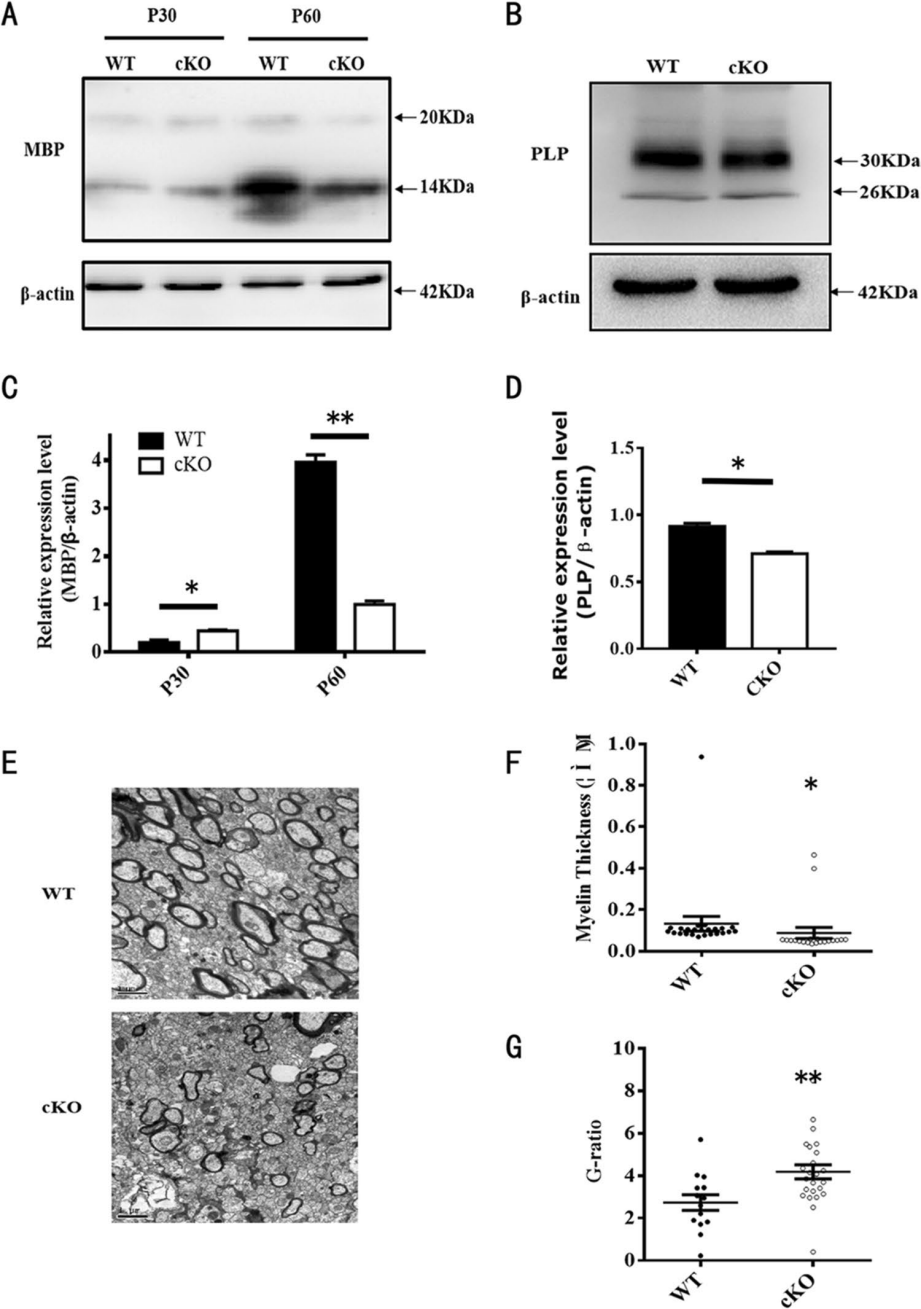
Deletion of ADAM10 in OPCs decreases the expression of myelin protein. (**A**) Detection of MBP proteins expression from the lysate of the whole brain at P30 and P60 in cKO and WT mice by Western blot. β-Actin blotting showed equal loading. (**B**) Detection of PLP proteins from the lysate of the whole brain at P60 in cKO and WT mice by Western blot. (**C**) Quantitative analysis of the 14 kDa MBP expression levels. *n* = 3 from each group. Data represent mean ± SEM, *p < 0.05. (**D**) Quantitative analysis of the30 kDa PLP expression levels. *n* = 3 from each group. Data represent mean ± SEM, *p < 0.05. (**E**) Representative electron micrographs of P60 corpus callosum from WT and ADAM10 cKO mice. Scale bar, 1 μm. (**F**) Quantification of the thickness of myelinated axons in defined areas from corpus callosum of P60 WT and ADAM10 cKO mice. *n* = 6 slides from 3 animals per group. Data were mean ± SEM, *p < 0.05. (**G**) Quantifification of G ratios for P60 corpus callosum in WT and ADAM10 cKO mice. *n* = 6 slides from 3 animals per group. Data were mean ± SEM, *p < 0.05

Consistent with the immunohistochemistry staining, western blotting showed that the level of MBP expression was also higher on P30 and lower on P60 in the ADAM10 cKO mouse brain (Fig. 6A, C). We also compared the expression levels of proteolipid protein (PLP), which accounts for approximately 50% of the myelin protein in adult CNS myelin. We found the same results as MBP in the corpus callosum of WT and ADAM10 cKO mice at P60 (Fig. 6C, D).

Electron microscopy (EM) showed that there was a significant reduction in the number of myelinated axons in the corpus callosum of ADAM10 cKO mice on P60 compared with WT mice (Fig. 6E). Moreover, compared with WT mice, myelinated axons were characterized by higher G ratios and thinner myelin sheaths in the corpus callosum of ADAM10 cKO mice (Fig. 6F, G). However, ultrastructural defects of myelin were not observed on P60 in adult ADAM10 cKO mice (Supplementary Fig. 1). Taken together, these results indicate that ADAM10 is essential for CNS myelination in mice.

### Conditional Deletion of ADAM10 Interfered with the Development of OPCs

**Fig. 7:**
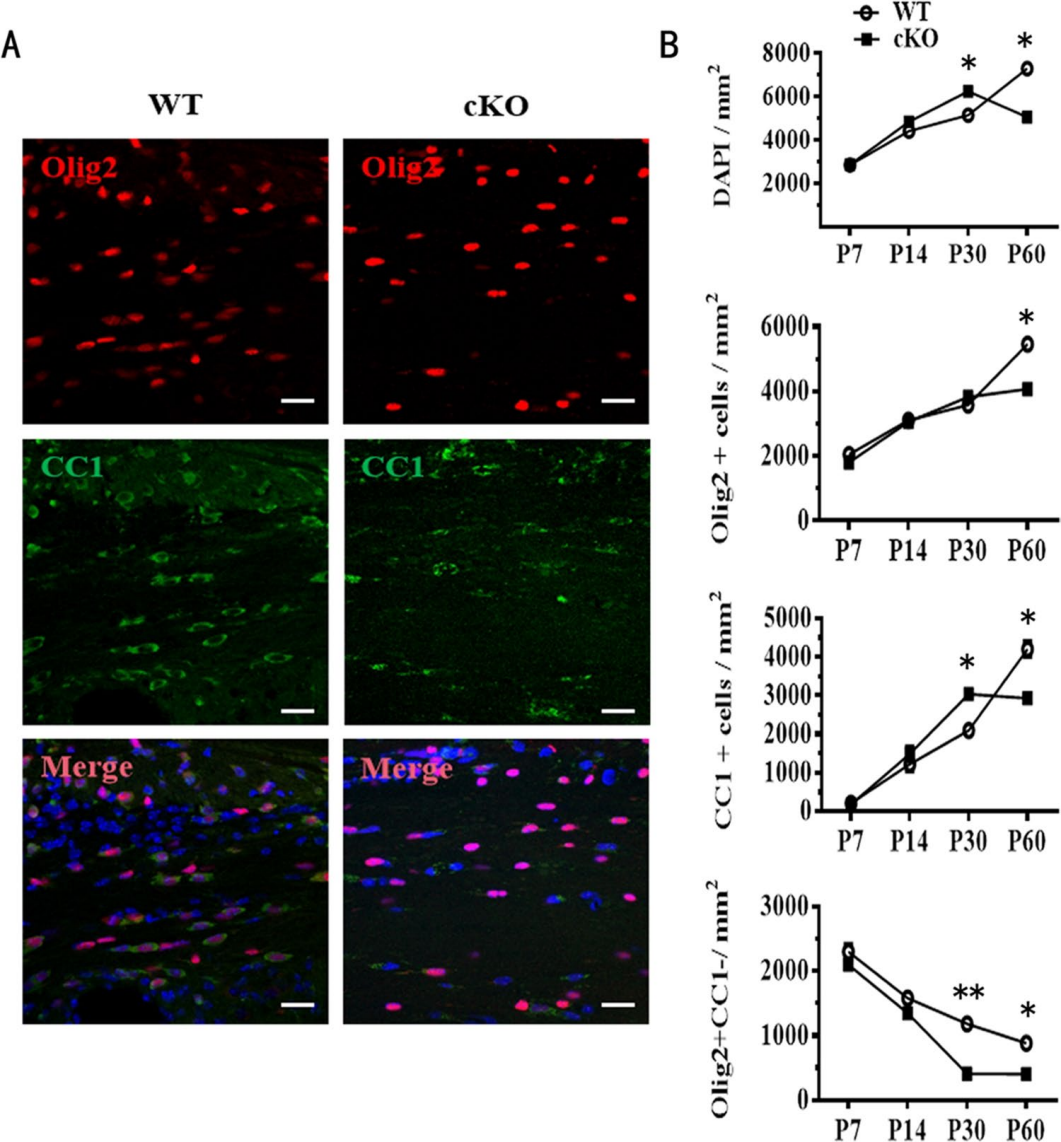
The number of OPCs and OLs is affected by conditional deletion of ADAM10 in OPCs. (**A**) Immunohistochemical analysis of coronal sections of the corpus callosum. Oligodendrocyte lineage cells and OLs were labeled with antibodies against Olig2 (red) and CC1 (green) respectively in the corpus callosum of P60 WT and ADAM10 cKO mice. Nuclei were counterstained with DAPI (blue). Scale bar: 20 μm. (**B**) Quantifification of cell types in the corpus callasum at P7, P14, P30, and P60. *n* = 3 slides from three animals per group at each time point, Data represent mean ± SEM, *p < 0.05. DAPI, 4, 6-diamidino-2-phenylindole dihydrochloride

Since dysmyelination implies fewer OPCs or mature OLs, we speculate that conditional deletion of ADAM10 in OPCs interferes with their development. Therefore, we examined the expression of Olig2 (a sustained marker for oligodendrocyte lineage cells from primary to mature OLs) and CC1 (a marker for post-mitotic OLs) in the corpus callosum of WT and ADAM10 cKO mice at P7, P14, P30, and P60 (Fig. 7A, B). We found that the density of Olig2 + cells was similar in the corpus callosum of the WT and ADAM10 cKO mice on P7, P14, and P30, but lower in ADAM10 cKO mice than that of WT mice on P60; the density of CC1 + cells continuously increased from P7 to P60 in the corpus callosum of WT mice, but it peaked on P30, and decreased on P60 in ADAM10 cKO mice. It is also worth noting that the density of Olig2 + /CC1- cells was reduced from P7 to P60 in both WT and ADAM10 cKO mice. However, the degree of decrease was greater at P30 and P60 in the corpus callosum of ADAM10 cKO mice than in WT mice, further demonstrating that the reduction of Olig2 + /CC1- OPC counts may be the reason for the difference in Olig2 + oligodendrocyte lineage cells in ADAM10 cKO mice.

**Fig. 8:**
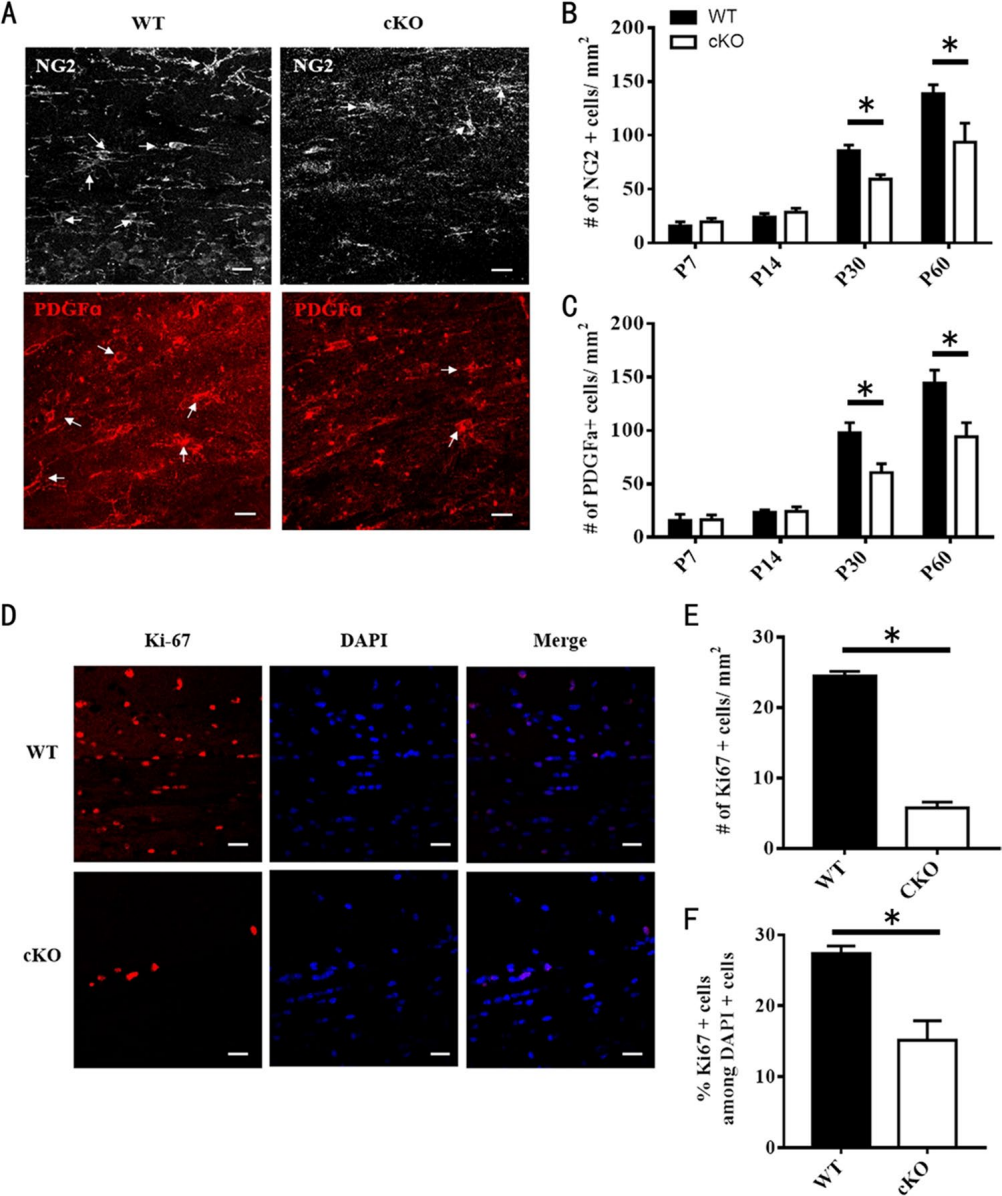
Proliferation of OPCs is inhibited by conditional deletion of ADAM10. (**A**) Immunohistochemical analysis of coronal sections of the corpus callosum from WT and ADAM10 cKO mice at P60. OPCs were labeled with antibodies against NG2 (white) and PDGFɑ (red) with highly branched morphology. Scale bar: 20 μm. (**B**, **C**) Quantifification of cell types in the corpus callosum at P7, P14, P30, and P60. *n* = 3 slides from three animals per group at each time point. Data represent mean ± SEM, *p < 0.05. (**D**) Coronal sections of P60 corpus callosum immunolabeled with a polyclonal antibody against Ki67 (red). Nuclei were counterstained with DAPI (blue). Scale bar: 20 μm. DAPI, 4, 6-diamidino-2-phenylindole dihydrochloride. (**E**, **F**) Quantification of the density of Ki67 + cells and percentage of KI67 + cells in the corpus callosum of WT and ADAM10 cKO mice at P60. *n* = 3 slides from three animals per group at each time point, Data represent mean ± SEM, *p < 0.05. Scale bar, 20 μm

We then examined the expression of NG2 and PDGFa, two OPC markers, in the corpus callosum of WT and ADAM10 cKO mice on P7, P14, P30, and P60 (Fig. 8A–C). We found no significant difference in the density of NG2 + and PDGFa + cells in the corpus callosum of WT and ADAM10 cKO mice on P7 and P14. However, at P30 and P60, the number of NG2 + and PDGFa + cells was lower in the corpus callosum of ADAM10 cKO mice than in WT mice, suggesting a downsized OPC pool in the brains of ADAM10 cKO mice. Next, we immunostained with an antibody against the cell cycle marker Ki-67 and found that the number of Ki67 + cells in the corpus callosum of ADAM10 cKO mice was statistically lower on P30 and P60 than that in WT mice (Fig. 8D–F), suggesting that the proliferation of NG2 + and PDGFa + OPCs was decreased in ADAM10 cKO mice.

Moreover, NG2 is not only expressed by OPCs but also by pericytes and neuronal precursor cells during development and adult; we also need to rule out the role of conditional knockout of ADAM10 in NG2 expressing cells on pericytes and other nerve cells in the brain. Therefore, we examined the expression of Laminin and Glucose Transporter 1 (GLUT1)—markers of vascular endothelium and basement membrane and the expression of GFAP, NeuN, and OX42—markers of astrocytes, neurons, and microglias by immunohistochemistry. We found that there was no difference on the vascular structure, the morphology and number of astrocytes, neurons, and microglias in the brain between ADAM10 cKO mice and WT mice at P60 (Supplementary Fig. 2, 3).

### Activation of Notch-1 Signaling Is Impaired in ADAM10 cKO Mice

**Fig. 9:**
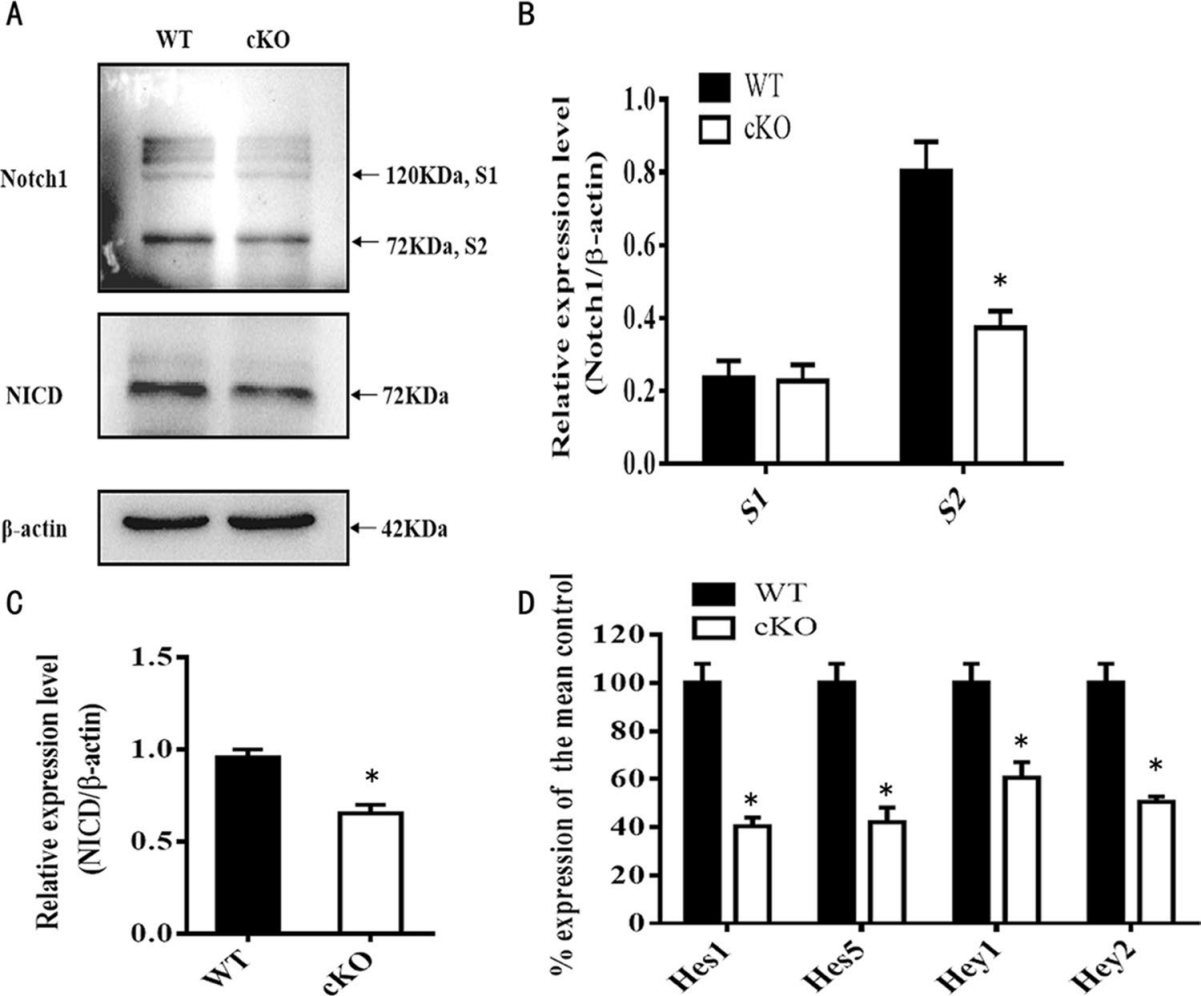
A defect in the proteolytic processing and regulation of Notch-1 signaling in brains of ADAM10 cKO mice. (**A**) Detection the expression of Notch1 and intracellular fragment of Notch-1 (NICD) with an anti-Notch-1mN1A antibody and an antibody against NICD from the lysate of the corpus collasum in ADAM10 cKO and WT mice at P60 by Western blot. β-Actin blotting showed equal loading. (**B**) Quantitative analysis of S1 and S2 Notch-1 cleavage products. *n* = 3 from each group, *p < 0.05. (**C**) Quantitative analysis of NICD fragments. *n* = 3 from each group, *p < 0.05. (**D**) qRT-PCR analysis of Notch-1 target genes Hes1, Hes5, Hey1, and Hey2 in the corpus collasum of WT and Adam10 cKO mice. β-Actin was used for normalization. *n* = 3 from each group, *p < 0.05

The similar abnormal CNS development between ADAM10 cKO and Notch-1 cKO mice [20] suggests that defects in myelination and OPC proliferation in ADAM10 cKO mice may be caused by inhibition of the Notch-1 signaling pathway. To investigate whether Notch-1 signaling is inactivated after ADAM10 conditional ablation in OPCs, we examined the expression of Notch-1 and Notch-1 fragments in the corpus collasum of WT and ADAM10 cKO mice by western blotting. We found that the Notch-1 S2 fragment (72 kDa) was significantly reduced in the corpus collasum of ADAM10 cKO mice, whereas the Notch-1 S1 cleavage product (120 kDa) was not significantly changed in the corpus collasum of ADAM10 cKO mice (Fig. 9A, B). We also immunoblotted extracts from the corpus collasum of WT and ADAM10 cKO mice, using an antibody directed against the Notch1 intracellular domain (NICD). We also found that there was a stronger decline in the generation of NICD in the corpus collasum of ADAM10 cKO mice than in WT mice (Fig. 9A, C).

To further demonstrate whether ADAM10 cKO affects the Notch-1 pathway in addition to altering Notch-1 expression, we used qRT–PCR to examine the expression levels of Hes1, Hes5, Hey1, and Hey2, which are the four major Notch-1 signaling downstream target genes. We found that levels of Hes1, Hes5, Hey1, and Hey2 expression were decreased by 60.1, 58.2, 38.9, and 51.4% in the corpus collasum of ADAM10 cKO mice, respectively (Fig. 9D), indicating that Notch-1 signaling was downregulated in the corpus collasum of ADAM10 cKO mice.

## Discussions

In the CNS, ADAM-10 mRNA was predominantly expressed in NeuN-positive neurons, not in GFAP-positive astrocytes, and was distributed in many restricted regions of the adult mouse brain, such as the cerebral cortex, hippocampus, thalamus, and cerebellar [21]. However, ADAM10 was widely expressed in the rat dorsal root ganglia (DRG) neurons as well as Schwann cells in PNS [12, 22]. Recently, ADAM10 was observed to be weakly expressed in oligodendrocytes (OLs) in numerous fiber tracts of the developing and adult brain, suggesting that ADAM10 may take part in the CNS myelination [23]. Oligodendrocyte progenitor cells (OPCs), also called NG2-glia, are a major type of glial cell and widely distributed in the adult CNS [24]. Under physiological conditions, they are the main source of myelinating OLs, and are regulated by a variety of signaling molecules that differentiate into OLs for the myelination of axons [3–5, 25]. ADAM10 has never been previously described to be expressed in OPCs in CNS, and it is still unclear, which extent OPCs express ADAM10 and how this expression may change during OPCs development and myelination in vivo. In this study, we first detected the expression pattern of ADAM10 in the white matter of the mouse brain using in situ hybridization (ISH) and immunohistochemical staining. Double staining results with ISH and immunohistochemistry demonstrated that the ADAM10 gene was weakly expressed in OPCs of the corpus callosum and hippocampus in the adult mouse brain.

ADAM10 is required for the control of embryonic development, neurogenesis, and axon extension in the CNS [26, 27]. ADAM10-deficient mice died on approximately embryonic day (E) 9.5 with severe developmental defects in the somites, heart, vasculature, and brain [9]. Conditional knockout of ADAM10 in neural progenitor cells (NPCs) leads to perinatal death with a disrupted neocortex and markedly reduced ganglionic eminence in mice [28]. Among known ADAMs, ADAM10 and ADAM17 (TNF-alpha converting enzyme (TACE)) have several structural and functional homologies [29]. ADAM17 plays a crucial role in OL expansion, cell cycle exit, and survival during postnatal myelination of the subcortical white matter (SCWM). ADAM10 is also essential for OL regeneration during CNS remyelination following demyelination by activating EGFR signaling in OL lineage cells [30, 31]. However, it is still unclear whether ADAM10 is involved in myelination of the CNS. Thus, we addressed the question of how ADAM10 regulates OPC development or myelination in vivo. In this study, we crossed NG2-Cre mice with ADAM10 ^loxp/loxp^ mice to generate mice with conditional knockout of ADAM10 in the OPC. It has been reported that the Cre of NG2-Cre mice is active in OPCs from the late embryonic stages (E14, Jackson Lab) throughout adulthood [15]. We firstly confirmed the recombination efficiency by using the ROSA26 reporter line in pilot studies and found that the NG2-cre transgenic mice expressed the most pronounced Cre recombinase 30 days after birth (Supplementary Fig. 4, 5). Consistently, ADAM10 cKO mice began exhibiting a significant body and behavioral abnormalities from postnatal (30 ± 2.1) day, and finally died at (65 ± 5) days after birth.

A series of behavioral analysis showed that ADAM10 cKO mice exhibited the obvious “anxiety and depression-like” performances before they died. Growing evidence has implicated neuroinflammation-induced demyelination as an important pathological feature in major depressive disorder (MDD) and multiple sclerosis (MS), and myelin deficits contribute to mood swing and cognitive decline [16, 17, 32]. Our results using FluoroMyelin Green fluorescent myelin staining, electron microscopy, immunohistochemistry, and western blotting demonstrated that deletion of ADAM10 in OPCs caused significant premyelination on P30 but demyelination on P60. In further studies, we found that conditional deletion of ADAM10 in OPCs interfered with the development of OPCs and decreased OPC proliferation in ADAM10 cKO mice. Moreover, we found that the number of OLs in ADAM10-knockout mice was reduced on P60, which may be related to the premature differentiation of OPCs and the resulting dysfunction and death of mature OL cells.

ADAM10 acts as a Notch sheddase and cleaves it at the S2 site in early and late embryos [33–35]. Generally, a negative regulatory region (NRR) locks down the Notch receptor in a protease-resistant state to protect the ADAM cleavage site. When a ligand-bearing cell binds to the Notch receptor, it induces a global conformational shift that unfolds the NRR structure to expose S2 cleavage ADAM’s site, [36] followed by γ-secretase cleavage to release the Notch-intracellular domain (NICD), which translocates to the nucleus and initiates transcription of Notch target genes with binding partners [37]. In the developing CNS, the function of Notch signaling routinely acts as “lateral inhibition” [38]. For example, when Notch-1 binds to its ligand Jagged1, it regulates oligodendrocyte differentiation and myelin formation by promoting OPC proliferation and restricting their maturation [39]. However, it is not unclear whether ADAM10 in OPCs regulates myelination through the Notch1 signaling in the developmental and adult mouse brain. Hence, we examined the expression of Notch-1 and proteolytic Notch-1 fragments by western blotting, and the expression of Notch-1 downstream of target genes Hes1, Hes5, Hey1, and Hey2 in the corpus collasum of WT and ADAM10 cKO mice. We found a significant decline in the generation of Notch-1 S2 fragments and NICD and the levels of the four Notch-1 target genes in the corpus collasum of ADAM10 cKO mice. This explains why NG2-positive, PDGFa-positive cells, and Ki67-positive proliferative OPCs decreased on P30 and P60 in this study, suggesting ADAM10 cKO in OPCs-induced impaired Notch-1 signaling activation, leading to the partial depletion of OPCs and premature differentiation. Notably, we found that there were more CC1 + OLs and myelination at P30, but fewer CC1 + OLs and myelination at P60 in ADAM10 cKO mice than in WT mice. If ADAM10 deficiency inhibits OPC proliferation and promotes OPC differentiation into OLs only through inactivation of Notch1 signaling, the number of OLs and myelinations should be increased in the brains of ADAM10 cKO mice. This indicates that ADAM10 in OPCs may also regulate CNS myelination and the survival and function of OPCs and OLs through other signaling pathways. To date, many substrates have been identified for catalytically active ADAM10, such as amyloid precursor protein (APP), Delta-like ligand-1 (Dll1), N-cadherin, and Fas-ligand [40–44].

In conclusion, our study showed that ADAM10 is also expressed in OPCs, and conditional knockout of ADAM10 in OPCs suppresses proliferation and promotes premature OPCs and premyelination through inactivation of the Notch-1 signaling pathway, inducing the inability of myelination maintenance and leading to “anxiety and depression-like” performance in mice. This study provides experimental evidence to demonstrate that ADAM10 is essential for modulating CNS myelination and OPC development by activating Notch-1 signaling in the developing and adult mouse brain. It is worth noting that Cre recombinase in NG2-Cre transgenic mice is under the control of the mouse Cspg4 (chondroitin sulfate proteoglycan 4) gene promoter, which is not only detected in OPCs, but also in pericytes. Therefore, these results may also be related to the knockdown of ADAM10 in pericytes.

## Supplementary Information

Below is the link to the electronic supplementary material.

Supplementary file1 (MP4 68039 KB)

Supplementary file2 (MP4 22338 KB)

Supplementary file3 (DOCX 2074 KB)

## Data Availability

The datasets used in this study are available from the corresponding author upon reasonable request.
