# Supplementary material for: A Disintegrin and Metalloproteinase 10 (ADAM10) Is Essential for Oligodendrocyte Precursor Development and Myelination in the Mouse Brain
Source: Mol Neurobiol. 2022 Dec 23;60(3):1675–89. doi: 10.1007/s12035-022-03163-0 (PMC9899191; doi:10.1007/s12035-022-03163-0)
Supplement: Supplementary file 3 — Supplementary file3 (DOCX 2074 KB) [file 12035_2022_3163_MOESM3_ESM.docx]

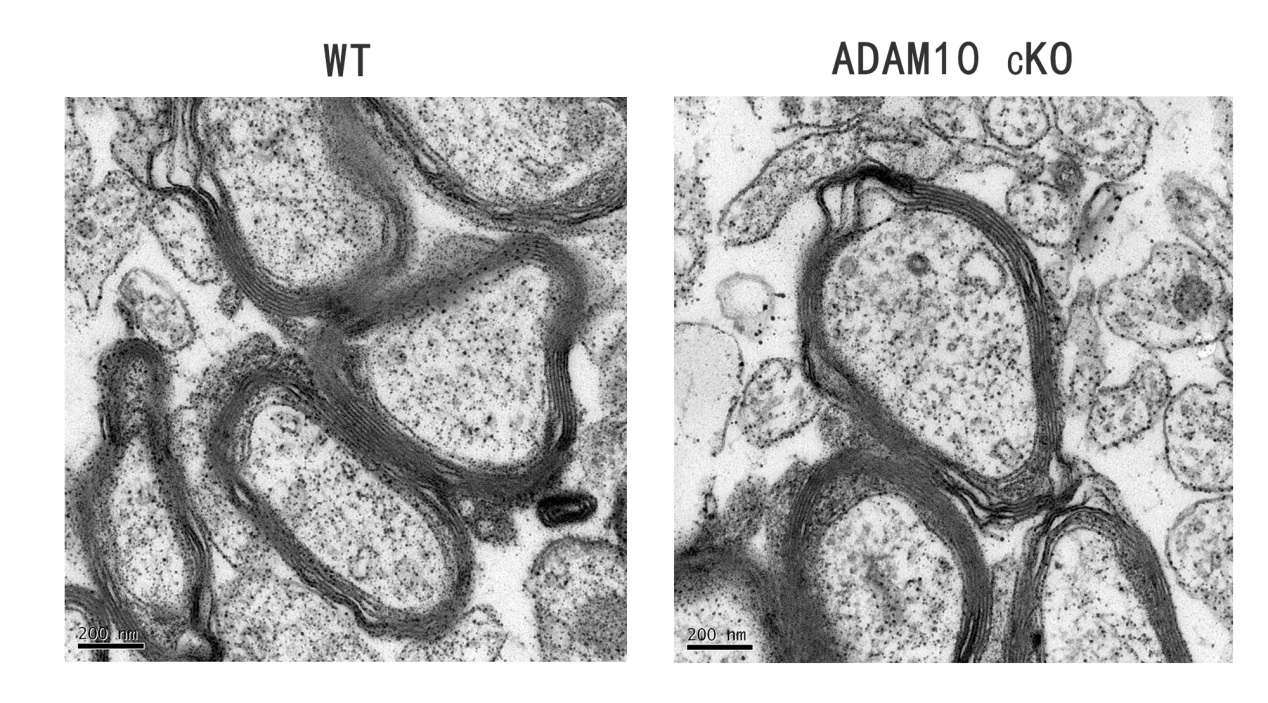


**Supplementary Figure 1.** Representative images of electron micrographs of corpus collasum from WT and ADAM10 cKO mice at P60.

**
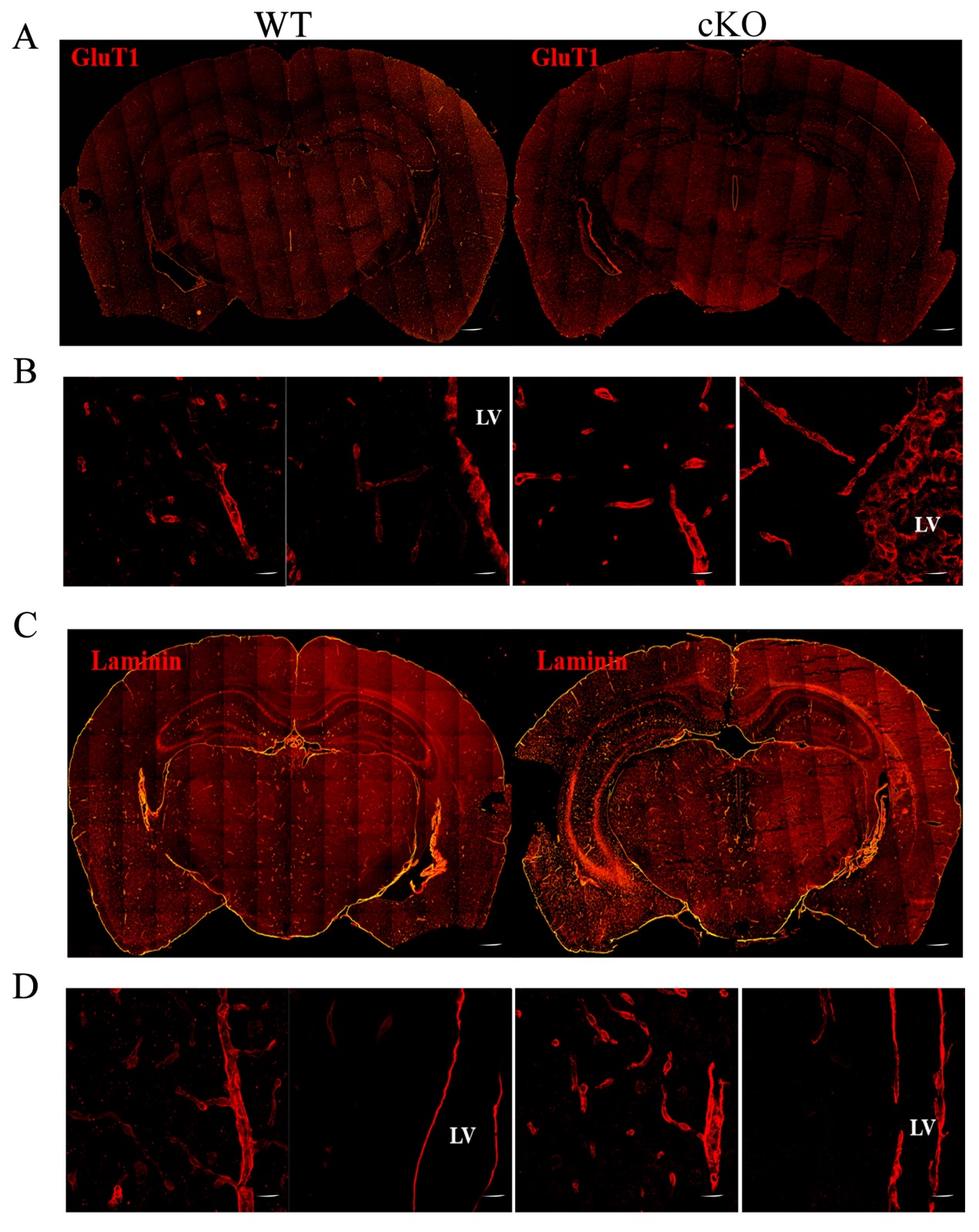
**

**Supplementary Figure 2. Deletion of ADAM10 in OPCs has no effect on Vascular structure in brain.** (A) Immunostaining of GLUT1 in brain at P60. LV, Lateral ventricle. (A) Scale bar: 200 μm. (B) Scale bar: 20 μm. (C) Immunostaining of Laminin in brain at P60. LV, Lateral ventricle. (A) Scale bar: 200 μm. (D) Scale bar: 20 μm.


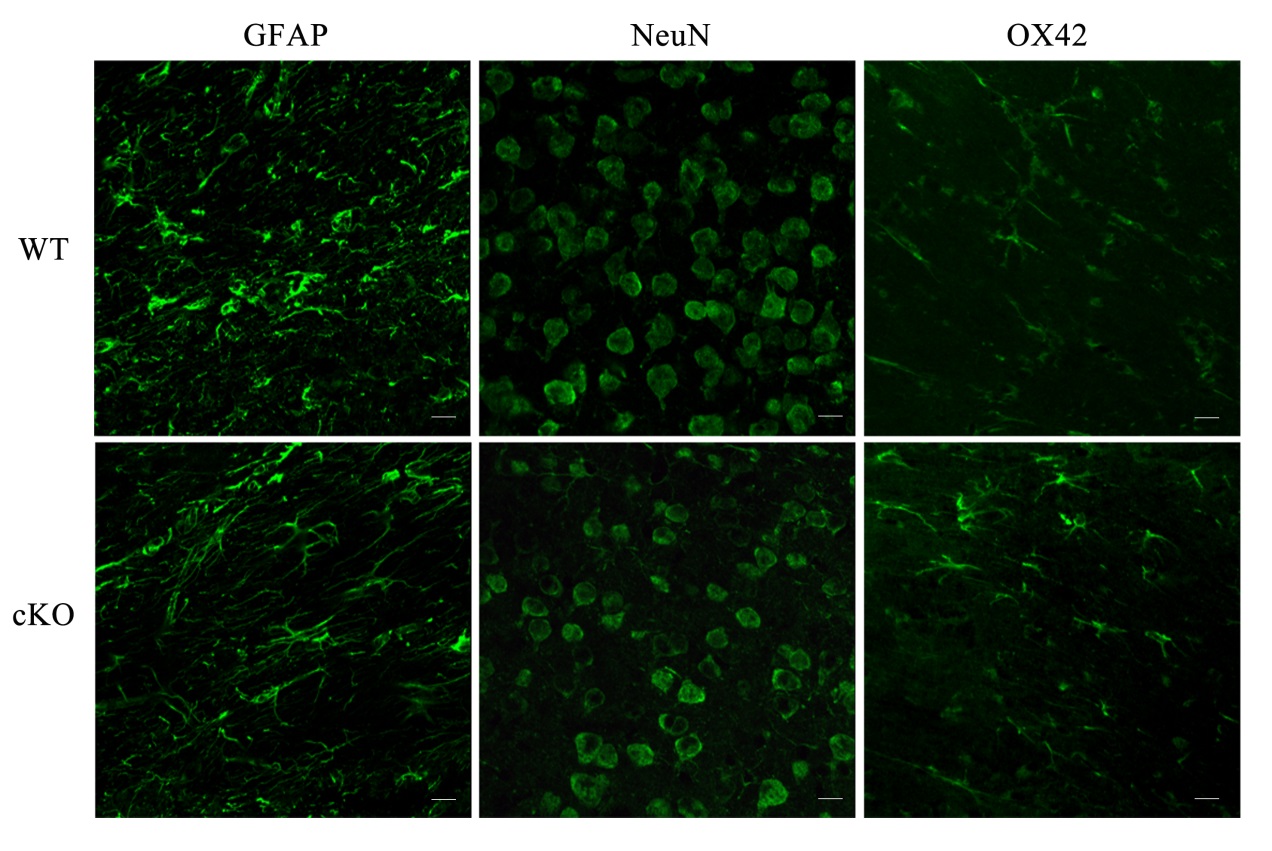


**Supplementary Figure 3. Deletion of ADAM10 in OPCs has no effect on the morphology and number of other nerve cells in brain.** (A) Immunostaining of GFAP in brain at P60. Scale bar: 20 μm. (B) Immunostaining of NeuN in brain at P60. Scale bar: 20 μm. (C) Immunostaining of OX42 in brain at P60. Scale bar: 20 μm.


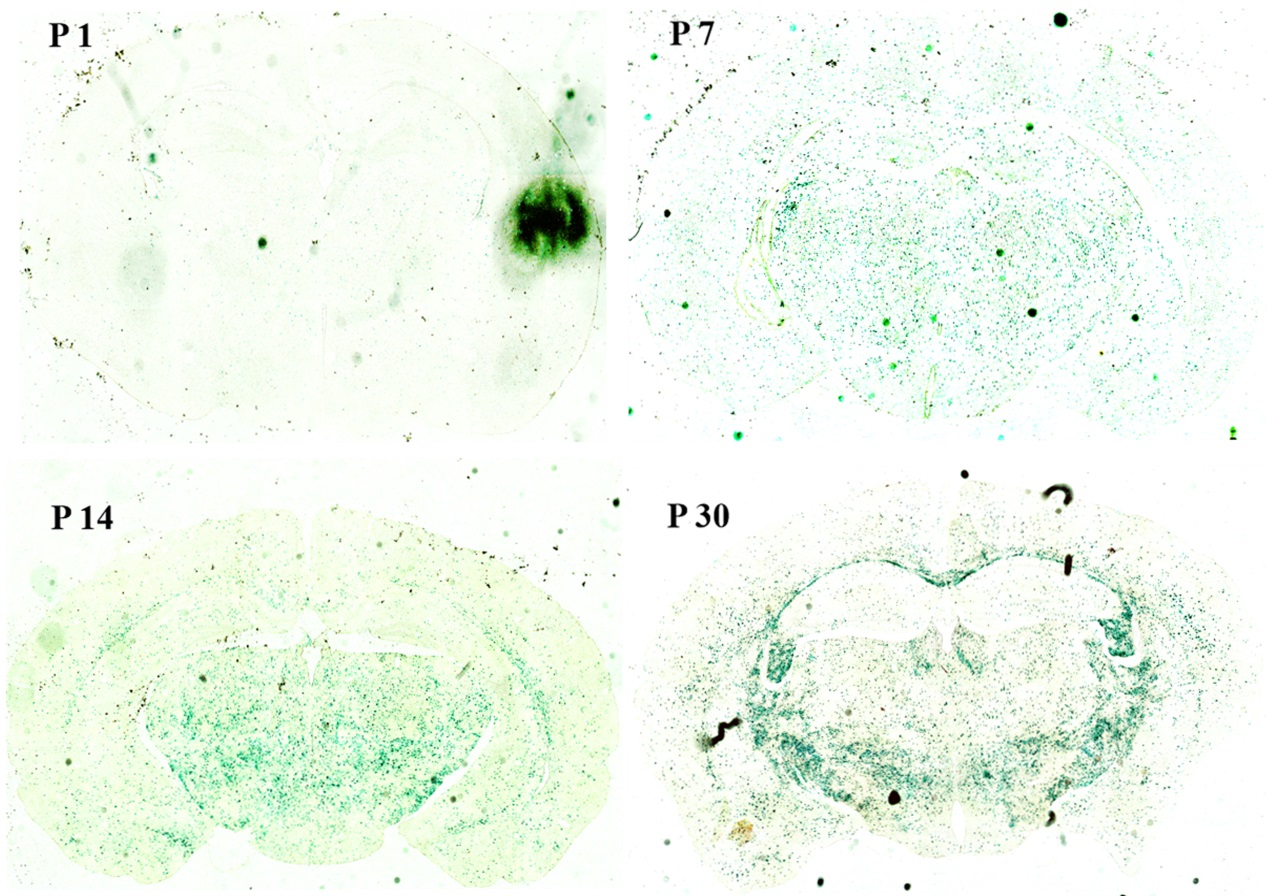


**Supplementary Figure 4.** NG2-cre transgenic mice express the most pronounced Cre recombinase at 30 days after birth

**
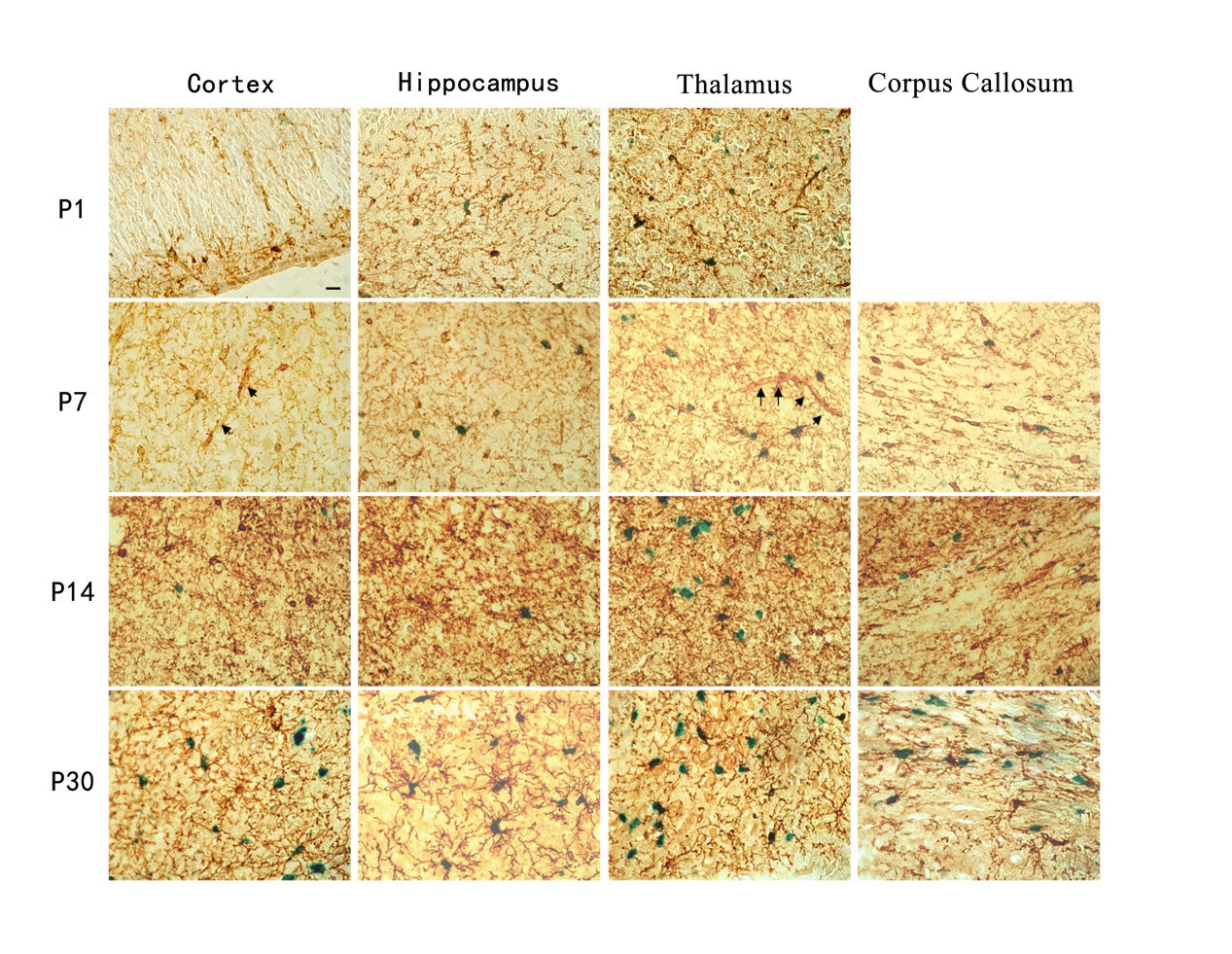
**

**Supplementary Figure 5. X-gal and NG2 immunohistochemical staining on the brain slices of the reporter mice at 1, 7, 14 and 30 days after birth.** All Cre were expressed in NG2+ OPCs (multiple branches), but not all NG2+ cells showed Cre activity. Cre was not expressed in NG2+ perivascular cells (around blood vessels) in the cortex, hippocampus, thalamus and corpus callosum of brain, suggesting that ADAM10 was specifically knocked out in OPCs in our experiments. Blue, X-gal; brown, NG2. Scale bar: 20 μm.
